# First-in-human safety and immunogenicity investigations of three adjuvanted reduced dose inactivated poliovirus vaccines (IPV-Al SSI) compared to full dose IPV Vaccine SSI when given as a booster vaccination to adolescents with a history of IPV vaccination at 3, 5, 12 months and 5 years of age

**DOI:** 10.1016/j.vaccine.2016.12.027

**Published:** 2017-01-23

**Authors:** Line M. Lindgren, Pernille N. Tingskov, Annette H. Justesen, Bettina S. Nedergaard, Klaus J. Olsen, Lars V. Andreasen, Ingrid Kromann, Charlotte Sørensen, Jes Dietrich, Birgit Thierry-Carstensen

**Affiliations:** a Statens Serum Institut, 5 Artillerivej, 2300 Copenhagen S., Denmark; b Center for Clinical and Basic Research (CCBR), Ballerup Byvej 222, 2750 Ballerup, Denmark; c CCBR Vejle, Orla Lehmannsgade 1^3^, 7100 Vejle, Denmark; d CCBR Aalborg, Hobrovej 42 D^2^, 9000 Aalborg, Denmark; e Larix A/S, Lyskær 8b, 2730 Herlev, Denmark

**Keywords:** Affordable IPV, IPV dose sparing, Dose investigation, Aluminium hydroxide adjuvant

## Abstract

**Background:**

There is a demand of affordable IPV in the World. Statens Serum Institut (SSI) has developed three reduced dose IPV formulations adsorbed to aluminium hydroxide; 1/3 IPV-Al, 1/5 IPV-Al and 1/10 IPV-Al SSI, and now report the results of the first investigations in humans.

**Methods:**

240 Danish adolescents, aged 10–15 years, and childhood vaccinated with IPV were booster vaccinated with 1/3 IPV-Al, 1/5 IPV-Al, 1/10 IPV-Al or IPV Vaccine SSI. The booster effects (GMTRs) of the three IPV-Al SSI were compared to IPV Vaccine SSI, and evaluated for non-inferiority.

**Immunogenicity results:**

The pre-vaccination GMTs were similar across the groups; 926 (type 1), 969 (type 2) and 846 (type 3) in the total trial population.

The GMTRs by poliovirus type and IPV formulation were:

Type 1: 17.0 (1/3 IPV-Al), 13.0 (1/5 IPV-Al), 7.1 (1/10 IPV-Al) and 42.2 (IPV Vaccine SSI).

Type 2: 12.5 (1/3 IPV-Al), 13.1 (1/5 IPV-Al), 7.6 (1/10 IPV-Al) and 47.8 (IPV Vaccine SSI).

Type 3: 14.5 (1/3 IPV-Al), 16.2 (1/5 IPV-Al), 8.9 (1/10 IPV-Al) and 62.4 (IPV Vaccine SSI)

Thus, the three IPV-Al formulations were highly immunogenic, but inferior to IPV Vaccine SSI, in this booster vaccination trial.

**Safety results:**

No SAE and no AE of severe intensity occurred. 59.2% of the subjects reported at least one AE. Injection site pain was the most frequent AE in all groups; from 24.6% to 43.3%. Injection site redness and swelling frequencies were < 5% in most and < 10% in all groups. The most frequent systemic AEs were fatigue (from 8.2% to 15.0%) and headache (from 15.0% to 28.3%). Most AEs were of mild intensity. In conclusion, the three IPV-Al SSI were safe in adolescents and the booster effects were satisfactory.

ClinicalTrials.gov registration number: NCT02280447.

## Highlights

• Three adjuvanted reduced dose IPV-Al SSI were safe and immunogenic in adolescents.
• The three IPV-Al SSI were highly immunogenic, but inferior to IPV Vaccine SSI as a booster.
• Reduced dose IPV-Al SSI is intended for markets in need of affordable IPV.

## 1 Introduction

The World Health Assembly launched the global polio eradication initiative (GPEI) in 1988, and as per the most recent strategic plan from 2012 [1], the last case of paralytic polio caused by wild poliovirus is expected to occur in the near future. Part of the GPEI is cessation of the use of oral polio vaccine (OPV) due to the risks of vaccine-associated paralytic polio (VAPP) and circulating vaccine-derived poliovirus (cVDPV). The first step is introduction of bivalent OPV (bOPV) which contains poliovirus types 1 and 3 only, and cessation of the use of trivalent OPV (tOPV), a process that is presently ongoing in the concerned countries. bOPV will accelerate cVDPV elimination, as most cVDPVs are type 2 (cVDPV2) [2], [3]. The fact that bOPV does not protect against type 2 poliovirus is partly justified by the last wild type 2 polio case dating back to 1999, and a historically low risk of cVDPV2, as a result of GPEI activities [4], [5], [6], [7]. The WHO recommends three doses of bOPV and one supplementary dose of inactivated polio vaccine (IPV), either with the first or last bOPV dose depending on country specific risk factors. The supplementary IPV will provide some protection against paralytic polio caused by wild type 2, cVDPV2 or VAPP [8]. Usage of bOPV is planned to end during 2019–20 [1] which will be the final step of complete cessation of all OPV use.

There is an increasing demand of affordable IPV in the countries affected by the above changes, and many initiatives are presently ongoing to meet this demand [3]. Statens Serum Institut (SSI) has developed three reduced dose IPV formulations by adsorption of the inactivated virus to aluminium hydroxide adjuvant, named; 1/3 IPV-Al SSI, 1/5 IPV-Al SSI and 1/10 IPV-Al SSI, and we now report the results of the first investigations of these vaccines in humans. Based on animal studies it was anticipated that up to 10 times reduction of the antigen doses of each of the three poliovirus types in IPV was feasible without compromising, to a clinically significant extent, the immunogenicity of the vaccine [9].

The safety and immunogenicity of IPV adsorbed to aluminium hydroxide are already well established through a long track record of worldwide clinical use in childhood combination vaccines [10], [11]. Based on existing pre-clinical toxicology studies, clinical trials and post-marketing experience with the marketed SSI vaccines; IPV Vaccine SSI [12], DTaP-IPV Vaccine SSI [13] and/or TdaP-IPV Vaccine SSI [14], no safety concerns were anticipated for the three new IPV-Al SSI formulations, before entering clinical development. Two dose investigation trials have now been conducted with the three new IPV-Al SSI formulations. In both trials, the primary objective was demonstration of the non-inferiority of the immunogenicity of the three new IPV-Al SSI in comparison to IPV Vaccine SSI. In the first dose investigation (proof of concept) trial, reported herein, the vaccines were for the first time administered to humans, given as a booster vaccination to Danish adolescents.

## 2 Materials and methods

Prior to initiation of the trial, the regional ethics committee of the capital region of Denmark (VEK No.: H-4-2014-087) and the competent authority, the Danish Health and Medicines Agency (DHMA No.: 2014071217), approved the clinical trial application (EudraCT No.: 2014-000052-29), including the informed consent form. The trial was conducted in accordance with good clinical practice (GCP) and the current version of the Declaration of Helsinki (adopted on the 64th WMA General Assembly, October 2013). The trial was registered under ClinicalTrials.gov registration number NCT02280447.

### 2.1 Trial design and subjects

The trial was a phase I/II, non-inferiority, observer-blinded, randomised and controlled trial, with three investigational IPV-Al SSI groups and one IPV Vaccine SSI comparator group. Before the first trial intervention, informed consent was obtained. For all groups, Visit 1 consisted of a pre-vaccination blood sample, one booster vaccination, and diary instructions. Visit 2, 28–35 days later, included a post-vaccination blood sample, diary review and recording of adverse events (AEs) and concomitant medications (CMs). The trial subjects were healthy, between 10 and 15 years old, and had completed primary infant vaccination with DTaP-IPV Vaccine SSI/Act-Hib at 3, 5 and 12 months of age and childhood booster vaccination with TdaP-IPV Vaccine SSI at 5 years of age in Denmark. Exclusion criteria were: vaccination with IPV ⩽ 5 years prior to inclusion or with OPV any time, vaccination with a live vaccine or treatment with systemic corticosteroids ⩽ 1 month prior to inclusion or treatment with immune modulating products ⩽ 3 months prior to inclusion. Three Center for Clinical and Basic Research (CCBR) investigational sites in Ballerup (site 1), Vejle (site 2) and Aalborg (site 3), all located in Denmark, recruited 3 × 80 trial subjects through advertisements placed in newspapers and electronic media, or posters in the local communities. The planned total number of 240 subjects were randomly allocated into the four groups with approx. 60 subjects per group. The trial consisted of two parts. In PART ONE, 50% (120) of the subjects were randomised with allocation weights 4:4:1:1 to IPV Vaccine SSI, 1/3 IPV-Al SSI, 1/5 IPV-Al SSI or 1/10 IPV-Al SSI, respectively. In PART TWO, the remaining 50% (120) were randomised with allocation weights 1:1:4:4. After completion of 50% (120) of the subjects, i.e. after completion of PART ONE, a planned midterm interim safety and immunogenicity analysis included 2 × 48 subjects from the IPV Vaccine SSI and 1/3 IPV-Al SSI groups. While this interim analysis was ongoing, the trial continued to recruit and randomise the remaining 50% (120) PART TWO subjects. The aim of the interim analysis in the trial was to conclude as early as possible if it was safe to continue an ongoing phase II dose investigation trial in infants. The observer-blinding of the trial ensured that only pre-specified unblinded study nurses and the trial monitor had access to the trial vaccines and the dispensing logs, and that only the unblinded study nurses were present during the administration of the trial vaccines. The remaining trial staff including the investigators assessing the adverse events, the clinical trial manager, and the laboratory staff at SSI performing the antibody determinations, were blinded until database lock and release.

### 2.2 Trial vaccines and administration

The trial vaccines were all stand-alone trivalent IPV containing inactivated poliovirus type 1 (Brunhilde), type 2 (MEF-1) and type 3 (Saukett). The declared amounts of the poliovirus types 1, 2 and 3 in the comparator vaccine, IPV Vaccine SSI, were 40, 8 and 32 D-antigen units (DU), respectively. These are standard doses used by all IPV manufacturers. In the three investigational reduced dose vaccines the corresponding declared amounts of types 1, 2 and 3 were: 1/3 IPV-Al SSI (13.3, 2.7 and 10.7 DU), 1/5 IPV-Al SSI (8, 1.6 and 6.4 DU) and 1/10 IPV-Al SSI (4, 0.8 and 3.2 DU), all formulations with aluminium hydroxide corresponding to 0.5 mg aluminium per vaccine dose (0.5 mL). The ratios between the three poliovirus types were the same as in IPV Vaccine SSI. The comparator, IPV Vaccine SSI, was a clear solution for injection where the three investigational, IPV-Al SSI, were suspensions for injection. The two formulations were visually distinguishable. One batch of each of the three investigational IPV-Al SSI was manufactured for use in the trial. All vaccines were stored between +2 °C and +8 °C at the investigational sites with monitoring of storage conditions. The four vaccines were administered as 0.5 mL intramuscular injections in the left deltoid muscle, by use of a syringe fitted with a 23 Gauge, 25 mm needle.

### 2.3 Randomisation methods

Randomisation lists were generated by a validated SAS program by a statistician at Larix A/S, who was not in any way involved in the data management or planning of the interim or final statistical analysis in the trial. These lists were kept in a restricted access folder and individually sealed randomisation envelopes were prepared in a restricted process, and distributed to the three CCBR investigational sites. The different randomisation weights during PART ONE and PART TWO, described above, were obtained by creating two randomisation lists and two sets of randomisation envelopes for each of the three sites. At 120 included subjects, all sites shifted to the PART TWO randomisation envelopes. At the sites, a new subject was allocated the lowest available subject (randomisation) number. By opening the randomisation envelope with the corresponding number, the unblinded staff responsible for allocation and/or administration of trial vaccine could identify the vaccine to be administered to the subject. This procedure applied for both PART ONE and PART TWO.

### 2.4 Safety assessments

**Fig. 1:**
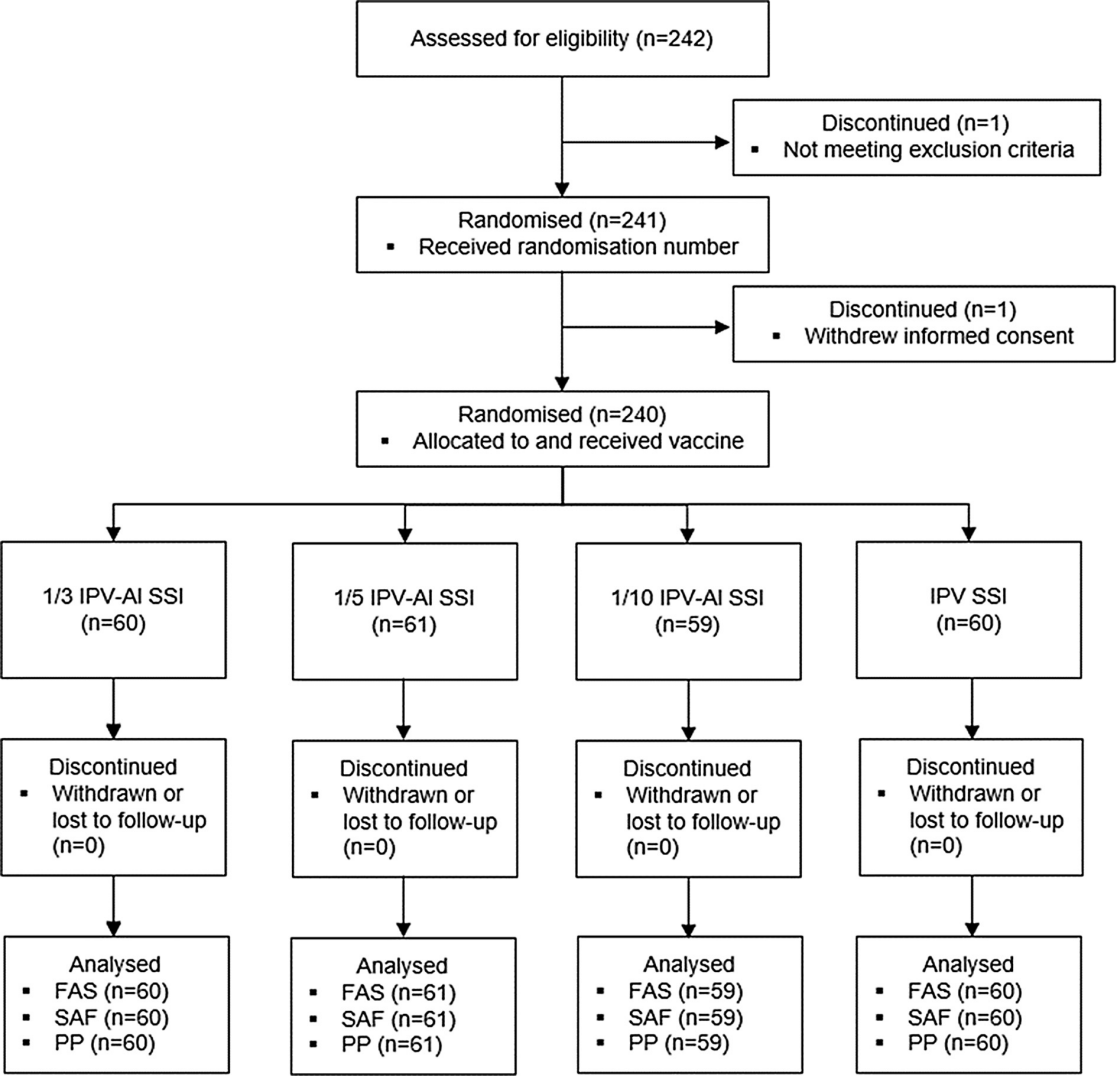
Disposition of subjects. The safety analysis set (SAF) was defined as randomised subjects who received a trial vaccination. The full analysis set (FAS) was defined as subjects who received a trial vaccination and had a post baseline immunogenicity measurement. The per protocol population (PP) was defined as the FAS with no major protocol deviations. All 240 subjects were included in the SAF, FAS and PP.

The subjects were observed for half an hour after the trial vaccination and immediate AE observations were recorded. A diary, thermometer and ruler were handed out to the subjects for daily recording and measuring of injection site reactions, temperature reactions and onset of other solicited AEs during the first three days (72 h) with follow-up and recording in the diary until resolved, and for recording of any AE, until the date of the follow-up at Visit 2 (28–35 days after Visit 1). The solicited adverse events in the diary were: Injection site redness or swelling reactions with a diameter ⩾ 25 mm, oral temperatures ⩾ 38.0 °C, other injection site reactions, nausea, vomiting, diarrhoea, headache, fatigue, myalgia. Concomitant medications (except for vitamins and minerals) were, furthermore, recorded. From 72 h after the vaccination until Visit 2 (28–35 days after the vaccination), the subjects were asked to fill in information in the diary, if they experienced an AE or if they took medication prescribed by a physician. All AEs in the diary were assessed for seriousness, relatedness to the vaccine, intensity and outcome, and were transferred to the eCRF by the investigators at Visit 2. Adverse events were coded by use of MedDRA, MSSO version 17.1. Concomitant medications were coded using the current version of the WHO Drug Dictionary Enhanced (WHO DDE) and tabulated by the anatomical therapeutic chemical (ATC) classification system. The safety results were analysed descriptively. The safety analysis set (SAF) was defined as randomised subjects who received the trial vaccination, Fig. 1.

### 2.5 Immunogenicity assessments

The Vero cell assay was used to determine the neutralising antibody against poliovirus types 1, 2 and 3, essentially as described in [15]. The assay was fully validated. Each well of two microtiter plates (96 wells) was filled with 50 μl of incubation medium. 50 μl of a serum sample was added to the first well in two rows on the first plate, and a two-fold dilution series was generated over two plates. Following this, approximately 100 CCID50 of poliovirus type 1, 2 or 3 in 50 μl incubation medium was added to each well, keeping just one polio type per plate. The microtiter plates were incubated on a shaker in an incubator at 37 °C ± 1 °C, 5% (4–6)% CO_2_ for 5½ (4–6) h. Afterwards the plates were incubated at 2-8 °C for 18–22 h. After incubation, 50 μl of Vero-cell suspension was added (60,000 cells/ml) to all wells in each plate. The plates were sealed with plate sealing tape and incubated at 37 °C ± 1 °C, 5 (4–6)% CO_2_ for 7 (6–8) days. The wells showing neutralising/cytopathogenic activity were recorded. The result from a single dilution series was given as 1/√2 times the lowest dilution factor with dead Vero-cells and the final titre calculated as the geometric mean of the results from the two independent dilution series. The immunogenicity of the investigated vaccines was assessed by calculation of geometric mean titres (GMTs) for polio antibody types 1, 2 and 3, before and after the vaccination, leading to the calculation of type 1, 2 and 3 GMT-ratios (GMTRs), referred to as booster effects. For each of the three IPV-Al SSI, the type-specific booster effects (GMTRs) were compared to that of the comparator, IPV Vaccine SSI, in the primary immunogenicity analysis, whereas in the secondary immunogenicity analyses type-specific seroconversion (⩾4 fold titre rise) rates (%) were compared. The full analysis set (FAS) was defined as subjects who received the trial vaccination and who had a post baseline immunogenicity measurement, where the per protocol population (PP) was defined as the FAS with no major protocol deviations, Fig. 1.

### 2.6 Statistical analysis

The primary endpoint was the booster effect defined as the day 28/day 0 titres. The sample size analysis for the primary endpoint was based on the following assumptions:

- A non-inferiority limit of −2 on the log2 scale, or 25% on the ratio scale
- 60 subjects per treatment group
- A one-sided alpha level of 2.5%
- Standard deviation on the log2-scale of 2.8, 2.2 and 3.5 (from [12]) for poliovirus types 1, 2 and 3
- No true mean difference between reduced dose and full dose of zero
- The overall power for one treatment against control was found as 85%.

With these assumptions, the combined power was 85% for rejecting H0 for each of the three poliovirus types. The primary endpoint was analysed on the log2 scale where the ratio day 28/day 0 titre corresponds to a difference since baseline. The ANCOVA model included treatment as factor and the pre-vaccination log2 titre as a covariate. All four treatments were included in the same model, but one model for each poliovirus type. The estimated treatment differences were transformed back and presented in tables as geometric mean titre ratios (GMTRs) = booster effects with 95% CI.

A non-inferiority criterion was used for concluding sufficient immunogenicity of each of the three IPV-Al SSI. Formulated on the ratio scale, the non-inferiority hypothesis was H0: Ti < 0.25 ∗ Tc, where Ti was the booster effect of one of the three investigational IPV-Al SSI and Tc was the booster effect of the comparator, IPV Vaccine SSI. Rejection of H0 meant that non-inferiority was concluded. For an IPV-Al SSI to be concluded non-inferior, H0 had to be rejected for each of the three poliovirus types. The booster effects of the three IPV-Al SSI were concluded independently. Since non-inferiority was only concluded for a given vaccine when it was demonstrated for all three poliovirus types, and since each of the three IPV-Al SSI were concluded independently, there was no need for multiplicity adjustment. SAS® version 9.3 was used for the statistical analysis.

## 3 Results

The trial subjects were recruited from 13 November 2014 to 5 March 2015. The midterm interim analysis conclusion was reached in May 2015 and the results of the final unblinded safety and immunogenicity analysis in September 2015.

### 3.1 Subject disposition and demographics

A total of 242 subjects were assessed for eligibility at Visit 1 of whom 240 were randomised and vaccinated: 1/3 IPV-Al SSI (60 subjects), 1/5 IPV-Al SSI (61 subjects), 1/10 IPV-Al SSI (59 subjects) and IPV Vaccine SSI (60 subjects), Fig. 1. As all subjects completed the trial and no major protocol deviations occurred, all subjects were included in the SAF, FAS and PP, Fig. 1. In the trial population of 10–15-years-old adolescents there were 43.8% (105/240) females and 56.3% (135/240) males. The mean age was 12.5 years. The age and sex distributions were similar across the groups.

### 3.2 Safety results

The conclusions of the midterm interim analysis of the first 120 included subjects were that there were no safety concerns, and that the ongoing phase II dose investigation trial in infants could continue as planned. The final safety analysis included a total of 240 subjects. No serious AE and no AEs of severe intensity occurred in the trial. A total of 59.2% (142/240) of the subjects reported at least one AE, with no major differences in frequencies between the groups. A total of 304 AEs were reported. Injection site pain was the most frequent injection site reaction (and the most frequent AE) in all groups with subject frequencies ranging from 24.6% to 43.3%. The frequencies of subjects with injection site redness and injection site swelling were < 5% in most and < 10% in all groups. No injection site redness or swelling reactions > 70 mm occurred. Three subjects experienced injection site redness ⩾ 50 mm, whereas five subjects experienced injection site swelling ⩾ 50 mm, Table 1. Two subjects, both from the IPV Vaccine SSI group, recorded pyrexia ⩾ 38.0 °C (38.10 °C and 38.20 °C) with onset within 72 h. The most frequent systemic AEs were fatigue and headache, with subject frequencies in the four groups of 10.0% (1/3 IPV-Al), 8.2% (1/5 IPV-Al), 8.5% (1/10 IPV-Al) and 15.0% (IPV Vaccine SSI) for fatigue, and 15.0% (1/3 IPV-Al), 18.0% (1/5 IPV-Al), 16.9% (1/10 IPV-Al) and 28.3% (IPV Vaccine SSI) for headache. Most of these AEs were of mild intensity. One subject experienced vaccine-related axillary pain, and one subject experienced vaccine-related lymphadenopathy.

### 3.3 Immunogenicity results

The final immunogenicity analysis included 240 subjects. At trial entry, all subjects were protected (titre ⩾ 8) against all poliovirus types except for two subjects (both with a titre ⩾ 4 for all poliovirus types). After the trial vaccination, all subjects in all groups had a titre ⩾ 8, and were thus protected against polio. The poliovirus type 1, 2 and 3 results are shown in Table 2, Table 3, Table 4. Before the booster vaccination (at Visit 1), the GMTs were similar across the groups, i.e. 926 (poliovirus type 1), 969 (poliovirus type 2) and 846 (poliovirus type 3) in the total trial population. After the booster vaccination (at Visit 2), there was a trend of a positive correlation between increasing post-vaccination GMTs and increasing antigen dose, Table 2, Table 3, Table 4.

**Fig. 2:**
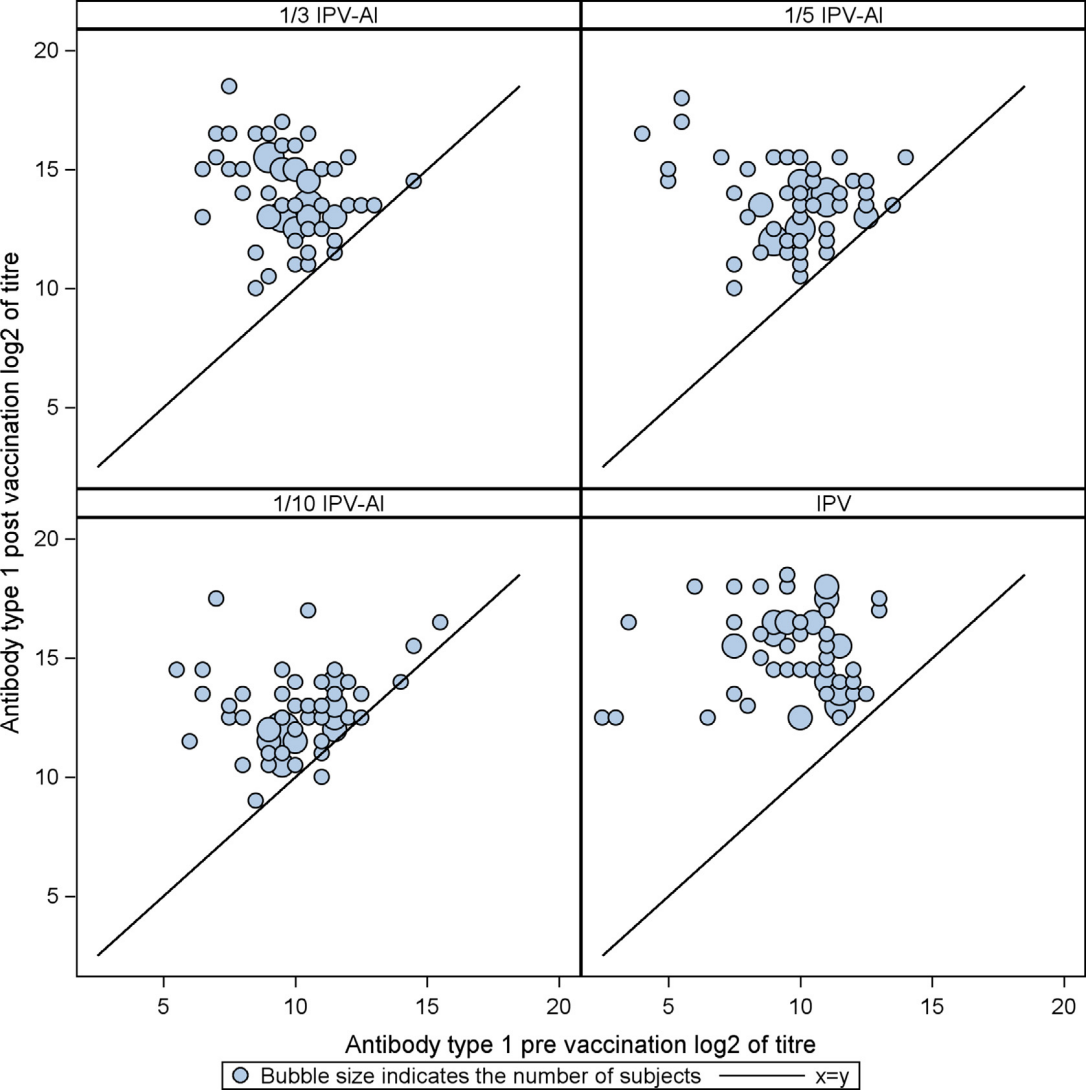
Scatter plots of poliovirus type 1 antibody log 2(titres) for 1/3 IPV-Al, 1/5 IPV-Al, 1/10 IPV-Al and the comparator IPV Vaccine SSI. The post-vaccination log 2(titre) of the individual subjects are plotted on the y-axis versus the pre-vaccination log 2(titre) on the x-axis. The seroprotection cut-off level (a titre ⩾ 8) in normal scale corresponds to a log 2(titres) ⩾ 3.

**Fig. 3:**
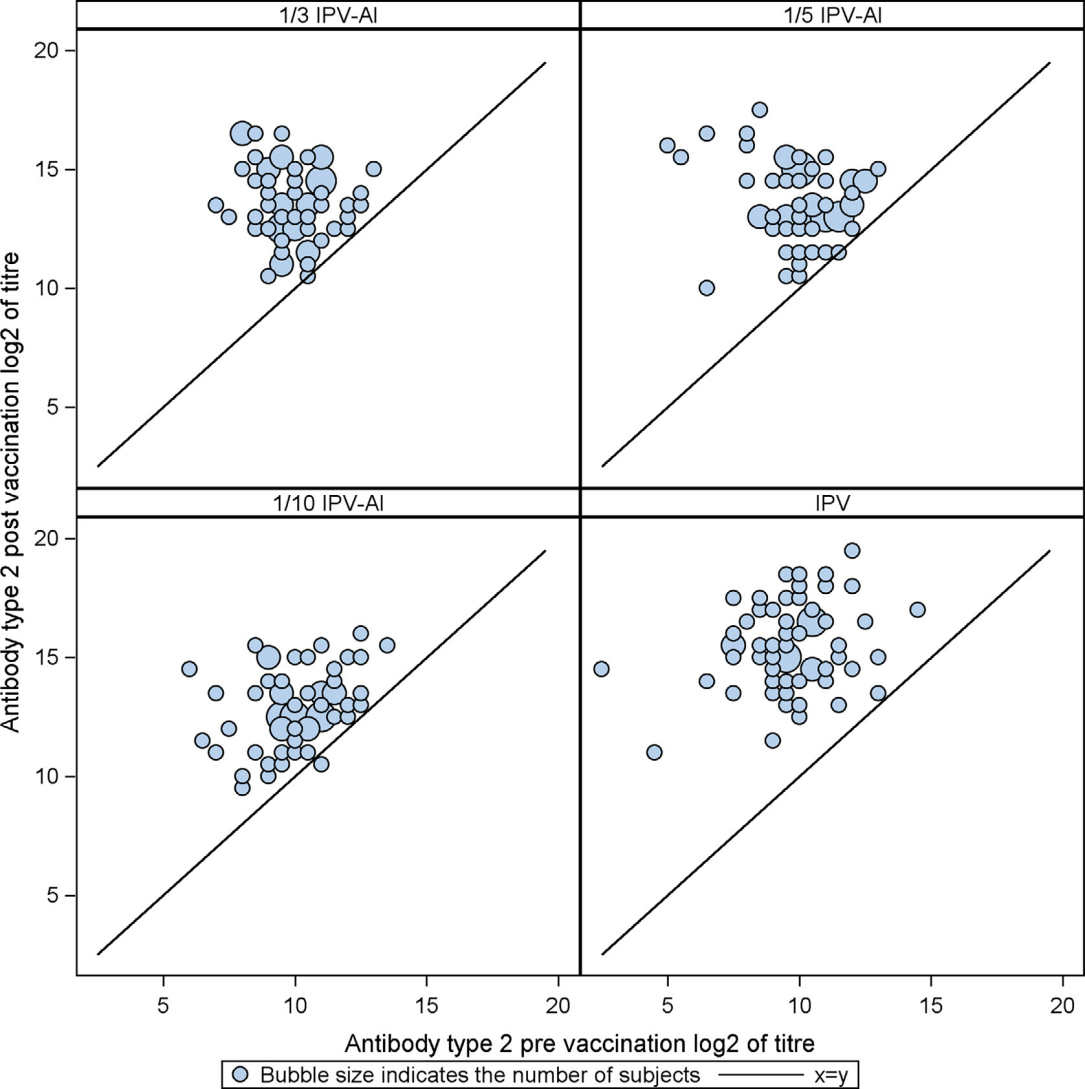
Scatter plots of poliovirus type 2 antibody log 2(titres) for 1/3 IPV-Al, 1/5 IPV-Al, 1/10 IPV-Al and the comparator IPV Vaccine SSI. The post-vaccination log 2(titre) of the individual subjects are plotted on the y-axis versus the pre-vaccination log 2(titre) on the x-axis. The seroprotection cut-off level (a titre ⩾ 8) in normal scale corresponds to a log 2(titres) ⩾ 3.

**Fig. 4:**
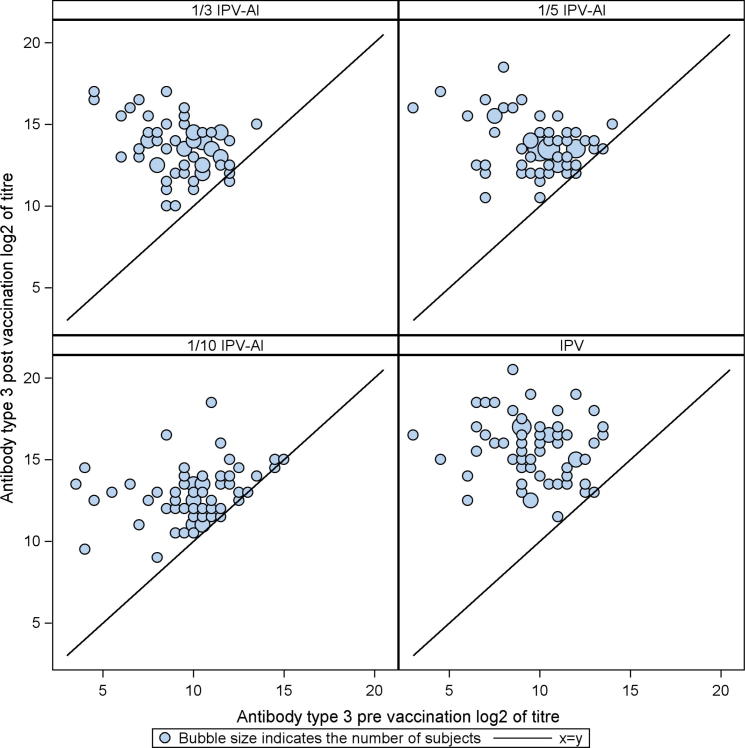
Scatter plots of poliovirus type 3 antibody log 2(titres) for 1/3 IPV-Al, 1/5 IPV-Al, 1/10 IPV-Al and the comparator IPV Vaccine SSI. The post-vaccination log 2(titre) of the individual subjects are plotted on the y-axis versus the pre-vaccination log 2(titre) on the x-axis. The seroprotection cut-off level (a titre ⩾ 8) in normal scale corresponds to a log 2(titres) ⩾ 3.

The booster effects (GMTRs) induced by the three reduced dose IPV-Al SSI and the comparator, IPV Vaccine SSI are shown in Table 2, Table 3, Table 4. For an IPV-Al SSI to be concluded non-inferior to IPV Vaccine SSI as a booster vaccine, the lower limits of the 95% confidence intervals (95% CI) were required to be ⩾ 0.25, for all poliovirus types. None of the three investigational IPV-Al SSI met these criteria. The scatter plots in Fig. 2, Fig. 3, Fig. 4 show the pre-vaccination versus the post-vaccination titres for the individual subjects, by poliovirus type and group. It is seen from the plots that the two subjects with a pre-vaccination log2titre < 3 (=a pre-vaccination titre < 8) for types 1 and 2, respectively, both elicited large booster responses after vaccination with IPV Vaccine SSI, Fig. 2, Fig. 3. All subjects had a pre-vaccination log2 titre ⩾ 3 (=a pre-vaccination titre ⩾ 8) for type 3. Across poliovirus types and trial groups it is, furthermore, seen that subjects with low pre-vaccination titres tend to have larger titre increases, indicated by the bubbles being more distant to the line at lower pre-vaccination titre values. Of note, the subjects with the lowest pre-vaccination titres, across poliovirus types, all had large titre increases even after vaccination with the lowest dose (1/10 IPV-Al SSI), Fig. 2, Fig. 3, Fig. 4.

## 4 Discussion

The target population for the IPV-Al SSI vaccine is infants in low resource countries where there is a need for increasing the availability of affordable IPV [2], [3], due to the ongoing switch from OPV to IPV as part of the progress of the GPEI in these countries [1]. Animal immunogenicity studies conducted by SSI [9] concluded that up to 10 times dose reduction of each of the three poliovirus types in IPV is possible in aluminium hydroxide adsorbed formulations. These studies motivated the development of three reduced dose IPV-Al SSI stand-alone formulations with 0.5 mg aluminium hydroxide to be investigated in humans. The overall aim of the present phase I/II first in human trial was to obtain initial results, to conclude whether an ongoing phase II dose investigation trial in infants with the same three reduced doses of IPV-Al SSI could continue as planned. The Danish 10–15 years old adolescents in the present trial had previously been vaccinated with IPV at 3, 5, 12 months (DTaP-IPV Vaccine SSI/Act-Hib) and 5 years (TdaP-IPV Vaccine SSI) of age. Thus, the trial vaccine was their fifth IPV vaccination. The comparator, IPV Vaccine SSI, is approved in several (nine) European countries for use in infants, children, adolescents and adults, for primary and booster vaccination against polio. IPV Vaccine SSI and other existing IPVs, e.g. non-adjuvanted stand-alone IPV, aluminium adjuvanted childhood combination vaccines with IPV, or booster combination vaccines with IPV, all contain the same amounts of 40, 8 and 32 D antigen units (DU), for poliovirus types 1, 2 and 3 [16].

The three IPV-Al SSI vaccines were safe and immunogenic. Non-inferiority to IPV Vaccine SSI could, however, not be demonstrated in the primary immunogenicity analysis, Table 2, Table 3, Table 4. Interestingly, a plain, non-adjuvanted, diphtheria and tetanus combination vaccine has previously been reported, to induce a more efficient (or more rapid) booster response in adults than a corresponding vaccine with aluminium hydroxide [17]. This may mean that the positive effect of aluminium hydroxide on IPV immunogenicity can only be demonstrated in a primary vaccination setting. Recently, a booster vaccination trial was conducted in French adolescents of 11–13 years of age with a similar history of IPV vaccination to the adolescents in our trial [18]. The administered approved and marketed DTaP-IPV in the French trial, i.e. a standard full dose IPV adsorbed to 0.3 mg aluminium hydroxide, resulted in booster effects (GMTRs) for poliovirus types 1, 2 and 3 ranging from 5.9 to 17.2 [18], where the booster effects of the three reduced dose IPV-Al SSI in the present trial ranged from 7.1 to 17.0, Table 2, Table 3, Table 4. This indicates that the booster effects of the three IPV-Al SSI investigated in the present trial are satisfactory, although inferior to plain non-adsorbed IPV.

At baseline all trial subjects in the present trial were protected (titre ⩾ 8) against all poliovirus types, except for two (both with a titre ⩾ 4 for all poliovirus types). After the booster vaccination, all subjects in all groups had a titre ⩾ 512 and were thus significantly above the protection limit of 8. Across poliovirus types and vaccination groups our results furthermore showed a trend that subjects with lower pre-vaccination titres exhibited larger titre increases following the booster vaccine, Fig. 2, Fig. 3, Fig. 4.

The pre-vaccination GMTs were approx. 900 across all poliovirus types and groups, Table 2, Table 3, Table 4. In two similar booster vaccination trials in French [18] and Canadian [19] adolescents, the pre-vaccination GMTs were considerably lower (below 500 for the three poliovirus types in both trials). Whether or not the relatively high pre-vaccination GMTs in the Danish adolescents had an influence on the magnitude of the booster responses is not clear. Of note, even if the Vero cell assay was used for determination of polio antibodies in all the trials discussed above, inter-trial comparisons of titre values or GMTs may not be possible due to inter-assay differences, whereas titre ratios (GMTRs) should be comparable. No serious or severe AEs occurred in the present trial. Injection site pain was the most frequent injection site reaction (and the most frequent AE) in all treatment groups, with subject frequencies ranging from 24.6% to 43.3%, Table 1. The frequencies of subjects with injection site redness or swelling were < 5% in most and < 10% in all groups, Table 1. No injection site redness or swelling reactions > 70 mm occurred. Thus, there were no indications that the aluminium hydroxide induced redness or swelling at the injection site. Injection site nodules have recently been estimated to occur in approx. 0.83% of infants completing a primary vaccination schedule with aluminium adjuvanted vaccines [20]. From the present trial with 240 subjects and a safety follow-up period of one month it is, unfortunately, not possible to report reliable frequencies of rare events and events with late onset, such as injection site nodules. The most frequent systemic AEs were fatigue and headache, with subject frequencies in the groups ranging from 8.2% to 15.0% for fatigue, and from 15.0% to 28.3% for headache. In conclusion, the three investigational IPV-Al SSI were safe and immunogenic in this first- in-human clinical trial in adolescents. This supported the continuation of further dose investigations in the target population of infants, and eventually the selection of one of the three doses of IPV-Al SSI for confirmatory phase III investigations in infants.

## Figures and Tables

**Table 1:** Subjects in the safety population (SAF) with injection site reactions or pyrexia, by intensity and group. N = total number of subjects in SAF, n = number of subjects with an AE,% = percentage of subjects with an AE, E = number of AEs. The AEs were coded by use of MedDRA MSSO version 17.1.

System organ class (SOC) *Preferred term (PT)*	1/3 IPV-Al	1/5 IPV-Al	1/10 IPV-Al	IPV Vaccine SSI	Total
	N = 60	N = 61	N = 59	N = 60	N = 240
	n (%) E	n (%) E	n (%) E	n (%) E	n (%) E
*General disorders and administration site conditions*					
Any mild AE	28 (46.7) 36	17 (27.9) 21	21 (35.6) 31	17 (28.3) 18	83 (34.6) 106
*Injection site pain*	25 (41.7) 25	14 (23.0) 14	18 (30.5) 20	15 (25.0) 15	72 (30.0) 74
*Injection site swelling*	2 (3.3) 2	2 (3.3) 2	4 (6.8) 4	1 (1.7) 1	9 (3.8) 9
*Injection site erythema*	3 (5.0) 3	1 (1.6) 1	4 (6.8) 4	1 (1.7) 1	9 (3.8) 9
*Injection site pruritus*	3 (5.0) 3	0	2 (3.4) 2	0	5 (2.1) 5
*Injection site haematoma*	1 (1.7) 1	2 (3.3) 2	1 (1.7) 1	0	4 (1.7) 4
*Injection site reaction*	0	2 (3.3) 2	0	0	2 (0.8) 2
*Injection site warmth*	1 (1.7) 1	0	0	0	1 (0.4) 1
*Axillary pain*	1 (1.7) 1	0	0	0	1 (0.4) 1
*Pyrexia*	0	0	0	1 (1.7) 1	1 (0.4) 1

*General disorders and administration site conditions*					
Any moderate AE	1 (1.7) 1	1 (1.6) 1	3 (5.1) 3	4 (6.7) 5	9 (3.8) 10
*Injection site pain*	1 (1.7) 1	1 (1.6) 1	1 (1.7) 1	2 (3.3) 2	5 (2.1) 5
*Injection site swelling*	0	0	1 (1.7) 1	1 (1.7) 1	2 (0.8) 2
*Injection site erythema*	0	0	0	1 (1.7) 1	1 (0.4) 1
*Injection site reaction*	0	0	1 (1.7) 1	0	1 (0.4) 1
*Pyrexia*	0	0	0	1 (1.7) 1	1 (0.4) 1

**Table 2:** Summary of poliovirus type 1 immunogenicity results in the per-protocol population (PP). N = total number of subjects in PP, n = number of subjects with data,% = percentage of subjects of PP, GMT = geometric mean titre, found as the 2^(mean(log 2(titres), GMTR = GMT ratio found as the ^(mean(log 2(titre 2/titre 1), [95% CI] = 2-sided 95% confidence interval for the geometric mean, based on the t-distribution with (n − 1) degrees of freedom for the log-transformed data. In the statistical analysis, ANCOVA, of the booster effect (GMTR) the change from baseline (visit 1) was analysed in a log transformed analysis with treatment and baseline value as factors. Antilog transformation yielded the baseline adjusted booster effect for each vaccine, and the ratio between the three investigational IPV-Al SSI and the comparator IPV Vaccine SSI.

Visit	Endpoint	1/3 IPV-Al N = 60	1/5 IPV-Al N = 61	1/10 IPV-Al N = 59	IPV Vaccine SSI N = 60	Total N = 240
	*Poliovirus type 1*					
Pre-vaccination Visit 1	GMT	901.8	878.4	1067.0	871.1	925.5
	[95% CI]	[679.8;1196.3]	[608.8;1267.4]	[747.6;1522;7]	[583.6;1300.1]	[779.0;1099.7]
	Seroprotection (Titre ⩾ 8)	100%	100%	100%	98.3%	99.6%
	Min; Max	90.5;23170.5	16.0; 16384.0	45.3;46341.0	5.7;8192.0	

Post-vaccination Visit 2	GMT	15734.8	12055.3	6553.1	39193.7	14894.6
	[95% CI]	[11443.1;21636.0]	[9083.3;15999.7]	[4851.8;8851.0]	[28388.3;54111.9]	[12566.0;17654.7]
	Seroprotection (Titre ⩾ 8)	100%	100%	100%	100%	100%
	Seroconversion (⩾ x4 Titre rise)	76.7%	80.3%	59.3%	90.0%	–
	Booster effect (GMTR)	17.0	13.0	7.1	42.2	–
	[95% CI]	[12.6;22.9]	[9.7;17.5]	[5.3;9.6]	[31.3;57.0]	–
	Booster effect ratio (GMTR_1/x IPV-Al_/GMTR_IPV SSI)_	0.402	0.308	0.169	–	–
	[95% CI]	[0.263;0.614]	[0.202;0.469]	[0.110;0.258]	–	–

**Table 3:** Summary of poliovirus type 2 immunogenicity results in the per-protocol population (PP). N = total number of subjects in PP, n = number of subjects with data,% = percentage of subjects of PP, GMT = geometric mean titre, found as the 2∗∗∗^(mean(log 2(titres), GMTR = GMT ratio found as the ^(mean(log 2(titre 2/titre 1), [95% CI] = 2-sided 95% confidence interval for the geometric mean, based on the t-distribution with (n-1) degrees of freedom for the log-transformed data. In the statistical analysis, ANCOVA, of the booster effect (GMTR) the change from baseline (visit 1) was analysed in a log transformed analysis with treatment and baseline value as factors. Antilog transformation yielded the baseline adjusted booster effect for each vaccine, and the ratio between the three investigational IPV-Al SSI and the comparator IPV Vaccine SSI.

Visit	Endpoint	1/3 IPV-Al N = 60	1/5 IPV-Al N = 61	1/10 IPV-Al N = 59	IPV Vaccine SSI N = 60	Total N = 240
	*Poliovirus type 2*					
Pre-vaccination Visit 1	GMT	972.1	1059.5	1060.7	808.1	969.3
	[95% CI]	[773.6;1221.6]	[793.3;1415.1]	[795.8;1413.9]	[569.4;1146.8]	[840.5;1118.0]
	Seroprotection (Titre ⩾ 8)	100%	100%	100%	98.3%	99.6%
	Min; Max	128.0;8192.0	32.0;8192.0	64.0;11585.2	5.7;23170.5	5.7;23170.5

Post-vaccination Visit 2	GMT	12133.2	12832.7	7457.1	45544.8	15198.8
	[95% CI]	[9162.2;16067.5]	[9640.8;17081.5]	[5602.4;9926.0]	[32835.4;63173.6]	[12860.2;17962.5]
	Seroprotection (Titre ⩾ 8)	100%	100%	100%	100%	100%
	Seroconversion (⩾×4 Titre rise)	76.7%	78.7%	67.8%	96.7%	–
	Booster effect (GMTR)	12.5	13.1	7.6	47.8	–
	[95% CI]	[9.4;16.7]	[9.9;17.5]	[5.7;10.2]	[35.8;63.8]	–
	Booster effect ratio (GMTR_1/x IPV-Al_/GMTR_IPV SSI)_	0.262	0.275	0.160	–	–
	[95% CI]	[0.174;0.394]	[0.183;0.413]	[0.106;0.241]	–	–

**Table 4:** Summary of poliovirus type 3 immunogenicity results in the per-protocol population (PP). N = total number of subjects in PP, n = number of subjects with data,% = percentage of subjects of PP, GMT = geometric mean titre, found as the 2^(mean(log 2(titres), GMTR = GMT ratio found as the ^(mean(log 2(titre 2/titre 1), [95% CI] = 2-sided 95% confidence interval for the geometric mean, based on the t-distribution with (n − 1) degrees of freedom for the log-transformed data. In the statistical analysis, ANCOVA, of the booster effect (GMTR) the change from baseline (visit 1) was analysed in a log transformed analysis with treatment and baseline value as factors. Antilog transformation yielded the baseline adjusted booster effect for each vaccine, and the ratio between the three investigational IPV-Al SSI and the comparator IPV Vaccine SSI.

Visit	Endpoint	1/3 IPV-Al N = 60	1/5 IPV-Al N = 61	1/10 IPV-Al N = 59	IPV Vaccine SSI N = 60	Total N = 240
	*Poliovirus type 3*					
Pre-vaccination Visit 1	GMT	652.6	951.1	1006.1	822.2	846.3
	[95% CI]	[464.1;917.6]	[649.9;1391.9]	[645.3;1568.7]	[549.6;1229.9]	[698.2;1025.8]
	Seroprotection (Titre ⩾ 8)	100%	100%	100%	100%	100%
	Min; Max	22.6;11585.2	8.0;16384.0	11.3;32768.0	8.0;11585.2	8.0;32768.0

Post-vaccination Visit 2	GMT	12488.7	13582.9	7457.1	52925.2	16125.8
	[95% CI]	[9346.0;16688.1]	[10271.0;17962.9]	[5492.4;10124.7]	[37447.7;74799.7]	[13535.2;19212.3]
	Seroprotection (Titre ⩾ 8)	100%	100%	100%	100%	100%
	Seroconversion (⩾×4 Titre rise)	78.3%	70.5%	59.3%	93.3%	–
	Booster effect (GMTR)	14.5	16.2	8.9	62.4	–
	[95% CI]	[10.7;19.5]	[12.0;21.8]	[6.6;12.1]	[46.3;84.1]	–
	Booster effect ratio (GMTR _1/x IPV-Al_/GMTR _IPV SSI)_	0.232	0.260	0.143	–	–
	[95% CI]	[0.152;0.354]	[0.170;0.395]	[0.094;0.219]	–	–
